# Palliative care provider attitudes toward existential distress and treatment with psychedelic-assisted therapies

**DOI:** 10.1186/s12904-021-00889-x

**Published:** 2021-12-20

**Authors:** Halsey Niles, Colleen Fogg, Ben Kelmendi, Mark Lazenby

**Affiliations:** 1 Massachusetts General Hospital, Boston, MA, USA; 2 Connecticut Mental Health Center, New Haven, CT USA; 3 Yale University School of Medicine, New Haven, CT USA; 4 University of Connecticut School of Nursing, Storrs, CT USA

**Keywords:** Psychedelic-assisted therapy, Existential distress, Spiritual distress, Demoralization, Psilocybin

## Abstract

**Background:**

Existential distress is a significant source of suffering for patients facing life-threatening illness. Psychedelic-Assisted Therapies (PAT) are novel treatments that have shown promise in treating existential distress, but openness to providing PAT may be limited by stigma surrounding psychedelics and the paucity of education regarding their medical use. How PAT might be integrated into existing treatments for existential distress within palliative care remains underexplored.

**Methods:**

The present study aimed to elucidate the attitudes of palliative care clinicians regarding treatments for existential distress, including PAT. We recruited palliative care physicians, advanced practice nurses, and spiritual and psychological care providers from multiple US sites using purposive and snowball sampling methods. Attitudes toward PAT were unknown prior to study involvement. Semi-structured interviews targeted at current approaches to existential distress and attitudes toward PAT were analyzed for thematic content.

**Results:**

Nineteen respondents (seven physicians, four advanced practice nurses, four chaplains, three social workers, and one psychologist) were interviewed. Identified themes were 1) Existential distress is a common experience that is frequently insufficiently treated within the current treatment framework; 2) Palliative care providers ultimately see existential distress as a psychosocial-spiritual problem that evades medicalized approaches; 3) Palliative care providers believe PAT hold promise for treating existential distress but that a stronger evidence base is needed; 4) Because PAT do not currently fit existing models of existential distress treatment, barriers remain.

**Conclusions:**

PAT is seen as a potentially powerful tool to treat refractory existential distress. Larger clinical trials and educational outreach are needed to clarify treatment targets and address safety concerns. Further work to adapt PAT to palliative care settings should emphasize collaboration with spiritual care as well as mental health providers and seek to address unresolved concerns about equitable access.

**Supplementary Information:**

The online version contains supplementary material available at 10.1186/s12904-021-00889-x.

## Background

Existential distress, which overlaps with the concepts of *existential suffering* [1], *spiritual distress* [2], *and demoralization* [3], is distress that arises for patients in the contemplation of their own mortality and is characterized by feelings of helplessness, loneliness, anxiety, and loss of meaning and purpose [4]. Existential distress has been widely recognized as a significant source of suffering for patients facing life-threatening illness (LTI) and can influence patients’ desire to hasten death or even take their own lives [5, 6]. Evidence-based treatments for existential distress remain limited: Pharmacological treatments for depressive symptoms are less effective in patients with LTI than in the general population [7] and targeted psychotherapeutic interventions demonstrate only modest benefit [8–13].

Psychedelic-assisted therapies (PAT), which apply psychotherapeutic approaches to altered states of consciousness produced by agents such as psilocybin, 3,4-Methylenedioxymethamphetamine (MDMA) or ketamine, may be potent treatments for patients facing existential distress in the setting of LTI. Randomized-controlled crossover trials using psilocybin have demonstrated reduced depression, anxiety and fear of death in patients with cancer- associated anxiety or depression [14–16], with benefits persisting in 60–80% of survivors at 6 month follow-up [14, 15]. Pilot trials with ketamine [17–19] and MDMA [20] suggest similar reductions in distress associated with LTI. While the evidence base still awaits larger confirmatory trials, these encouraging phase 2 results have brought discussions of psychedelic medicine into mainstream circles [21, 22] and generated proponents within palliative care [23, 24]. At the same time, stigma around psychedelics persists [25] despite their benign safety profile compared to similarly classified controlled substances [26, 27].

Whether PAT become broadly implemented within palliative care will depend not only upon the results of larger clinical trials but also the attitudes of health care providers in a position to recommend PAT. Qualitative studies of palliative care providers have identified key themes regarding attitudes toward the use of PAT in palliative settings [28, 29], as well as insights into how existential distress arises [30] and is identified by palliative care nurses [31, 32]. However, there has been little exploration into how PAT are perceived within the broader context of existential distress treatments provided by the multidisciplinary palliative care workforce. In order to provide context and direction to future work with PAT in patients with LTI, we conducted a qualitative study of palliative care providers to elicit themes regarding 1) their attitudes toward current treatments for existential distress and 2) the potential of PAT to treat existential distress associated with LTI.

## Methods

### Study overview and setting

We conducted qualitative semi-structured interviews with palliative care physicians, advanced practice nurses, chaplains, psychologists, and social workers currently working in palliative care settings within the United States.

### Sample and recruitment

Participants were recruited via email between May 2019 and August 2020 using a combination of purposive and snowball sampling methods. Participants were not selected for knowledge about or known positions regarding PAT. Participants provided informed consent and were not directly compensated; one randomly-selected participant received an online gift card after study completion. Recruitment continued until interviews failed to reveal significant new thematic content.

### Interview guide

The interview guide was developed by an interdisciplinary team including experts in palliative care (M.L.) and PAT (B.K.) and was directed at two key research questions: (1) How do palliative care providers view their role regarding existential distress and its treatment? (2) What are their attitudes toward PAT as potential treatments for existential distress in LTI? The final interview guide is available in Supplementary Materials.

### Data collection

Recorded interviews were conducted by H.N. via phone, Zoom video conferencing software, or in person. Interviews ranged in duration from 32 to 52 min, and concluded once participants had responded to all sections of the semi-structured guide and had been offered the chance to extrapolate further on earlier responses. Recorded audio from each interview was transcribed using Microsoft Word and de-identified prior to qualitative analysis. After the interview, participants completed an online survey covering basic demographics, clinical setting, and health care experience.

### Data analysis

Transcriptions were uploaded to Dedoose software version 8.3 and coded using a grounded theory approach [33, 34]. Initial themes were developed through open coding by M.L. and H.N., then applied to subsequent interviews by H.N and C.F. and updated in an iterative fashion following discussion and agreement of team members. Coders met regularly to resolve discrepancies and ensure adequate inter-rater agreement. Once coding was complete, study team members met to discuss grouping of codes and finalize key themes within the data [34].

### Thematic validation

Themes, subthemes, and representative quotations were then sent to a subset of 5 participants for validation. Criteria for validation was ≥.78 agreement for each individual theme and subtheme.

## Results

### Study sample

Thematic saturation was achieved after 19 interviews. Table 1 describes participants’ demographic characteristics. No participants reported prior clinical experience with PAT. Two participants reported no prior awareness of PAT.

**Table 1:** Participant demographics

	n (%)
**Gender**	
Female	13 (68)
Male	6 (32)
**Race**	
Caucasian	18 (95)
Black or African-American	1 (5)
**Ethnicity**	
Not Hispanic/Latinx	19 (100)
Hispanic/Latinx	0 (0)
**Age**	
***≤*** 35	3 (16)
36–50	8 (42)
50–65	6 (32)
66 ***≤***	2 (11)
**Profession**	
Chaplain	4 (21)
Nurse Practitioner	4 (21)
Physician	7 (37)
Psychologist^a^	1 (5)
Social Worker^a^	3 (16)
**Setting**	
Academic Medical Center	15 (79)
Small outpatient clinic/in-home services	4 (21)
**U.S. Region**	
Northeast	10 (53)
South	3 (16)
West Coast	6 (32)
**Primary Role**	
Clinician	16 (84)
Educator or Researcher	3 (16)
**Years working in Palliative/Hospice Care**	
< 5	10 (53)
5–9	3 (16)
10–14	3 (16)
15–19	3 (16)

^a^Hereafter grouped together to preserve confidentiality of responses

### Thematic analysis

**Fig. 1:**
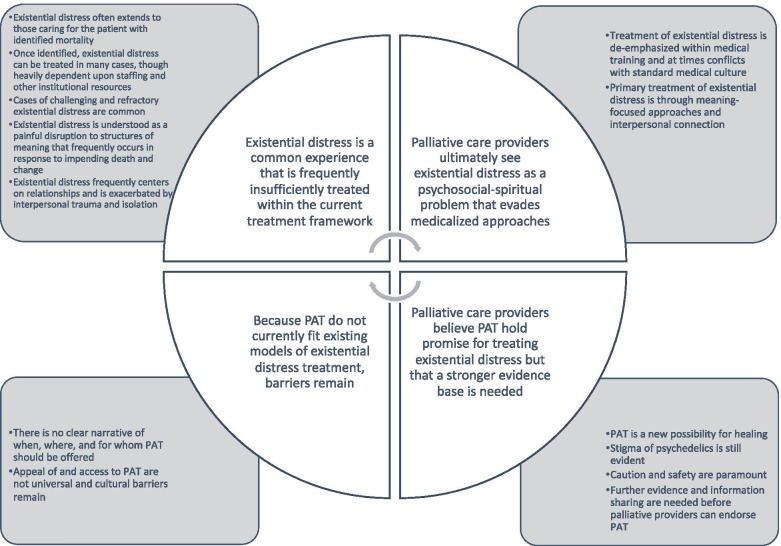
Themes and Subthemes

Four major themes and 15 subthemes were identified. All major themes and 13 subthemes met validation criteria, and are featured in Fig. 1. Validated themes with example quotations are displayed in Table 2.

**Table 2:** Identified themes and subthemes with representative quotations

Theme/Subtheme	Representative Quotations
**Theme 1: Existential distress is a common experience that is frequently insufficiently treated within the current treatment framework**	
Existential distress is understood as a painful disruption to structures of meaning that frequently occurs in response to impending death and change	*“[S]piritual or existential distress – which I look at as a break with your normal way of understanding yourself and the world; and relationship to the world; and the relationship, it could be to the transcendent, it could be to unity consciousness, it could be to God, but doesn’t have to be.”* *(S13, chaplain)*
	*“I would say most of my patients, in some way or another, are dealing with some kind of existential issues. Some are more distressed than others, but since most are facing mortality, these questions of an existential nature certainly come up, when reviewing one’s life, or when trying to think of what’s most important now, and how to continue to live a life that has meaning and purpose when facing mortality… I guess I become aware of it when patients are expressing a fear of death, a sense of…a sense of despair about their life, a loss of meaning, a loss of purpose. So it’s that kind of combination of despair, some hopelessness, uncertainty and fear of, particularly about, death that I consider as falling into that existential realm.”* *(S07, social worker/psychologist)*
	*“For someone who has had a diagnosis of Parkinson’s, or ALS, or cancer, or late-stage diabetes, or, you know, some of these god-awful things we’re dealing with, you know, the whole – the rug has been pulled out from under their lives, and now they’re facing financial changes and family changes and it’s just overwhelming. And that’s a total existential crisis. They’ve lost their vocation, they’re losing their mobility. It’s like ‘who am I?’”* *(S08, chaplain)*
	*“I think of it as somewhere between spiritual, meaning, kind of like larger distress issues. Like, separate from agitation, but you know spiritual, meaning, familial, kind of all wrapped into one. Separate from depression. Separate from agitation and delirium. But sometimes certainly contributing to all of the above.”* *(S01, physician)*
Existential distress frequently centers on relationships and is exacerbated by interpersonal trauma and isolation	*“So, like patients who have pain crises every time their spouse is there, or… thinking about disruptive relationships with grown children: there’s certain topics that come up as patients near end of life, like regrets about leaving a family, or not mending ties.”* *(S01, physician)*
	*“I would say patients who are unfriended, who might not have family or friends, or have other, you know, other core – whether a psychiatric diagnosis, or they have other types of trauma in their life: that might add an additional layer of complexity.”* *(S14, nurse practitioner)*
	*“For example, is there any unfinished business in their life? Is there some forgiveness that needs to be asked for? Do they need to forgive somebody else, is there a ruptured relationship from their past that needs to have a sense of closure, re-communion?”* *(S06, physician)*
	*“I used to work at the VA. I was at the VA for 5 years on their inpatient skilled nursing and palliative care team. And that, I felt like, was a different beast, where more than like 50%, probably more on the order of 75%, of those guys had some element of existential suffering. And those life experiences are very different. The population we work with in Palo Alto tends to be, you know, well educated; they have resources at their disposal. And it’s not to say that those things are protective from life experience, but they seem – they can help, in terms of the things that you’re sort of surrounded with, and your access to technology, right – to be able to like connect with your friends and family across the country if you’re alone – or having a caregiver who can help provide just, like, a touch every day.”* *(S16, nurse practitioner)*
Existential distress often extends to those caring for the patient with identified mortality	*“Families dealing with a lot of conflict, a lot of guilt over, a lot of caregiver burden and compassion fatigue: they also go through existential distress around broken dreams, around, uh, “I’ve lost my career. I never thought I was going to spend 10 years of my life doing this. Who am I now that they’re dying? I’m not ready to let them die because I’ve been a caregiver now for 12 years; what else am I going to do? I’m scared, I don’t know.” So it’s a family system, uh, journey, and our unit of care is the patient and family.”* *(S08, chaplain)*
	*“I think you have to learn the skill of addressing your own existential distress, because it does pop up… it’s important not to lose yourself in the work and in helping other people with their distress, because if you’re not, if you don’t find out your own way of coping, you will absorb it until you end up being the one with the distress.”* *(S15, social worker/psychologist)*
	*“[I]n the hospital setting, and in the general medical setting, we are horrible – oh wait, no, that’s not true – we are wonderful, we are starting to really recognize it, over and over again. Which actually creates distress because we can’t treat it. But we are just at the tip of the iceberg about how do we, transdisciplinarily, treat and address existential distress. Which includes our own existential distress that we can’t actually make it better.”* *(S13, chaplain)*
Once identified, existential distress can be treated in many cases, though heavily dependent upon staffing and other institutional resources	*“Well, I think we could always use more funding for additional staff and things like that… I mean, I’m within [dedicated cancer hospital], so you know we have more resources [here], yeah. More comprehensive like integrated medicine services, we have a dedicated chaplain. So, you know, compared to [general hospital], our patients are probably more supported in terms of resources for existential distress.”* *(S01, physician)*
	*“I work at a pretty impoverished small cancer center, and so we don’t have a lot of the great resources that giant, well-funded places that I’ve been before, like Dana-Farber where you can give reiki or acupuncture or Dignity Therapy and all these great things that are just wonderful because there’s a lot of resources and money. And we just don’t have any of that.”* *(S05, physician)*
	*“Ideally I think that I could probably – we could probably – be doing a bunch more, but we have a small team with limited resources, I guess is the bottom line.”* *(S10, social worker/psychologist)*
Cases of challenging and refractory existential distress are common	*“…other patients I think might not, um, might not have those supports or might not respond as well to the, you know, the quote-unquote treatments. For example, I think patients that, you know are typically younger: you know, when I see patients that are in their 30s or early 40s, you know with young kids, those patients that I feel like are more difficult to support, who might have, you know, distress that is not as easily addressed by our team”* *(S14, nurse practitioner)*
	*“I mean those are some of the other medications that we can use. Um, it doesn’t really help. I don’t know, I mean everybody’s different. I mean, I think sometimes existential distress can be a symptom of depression – I would look at it, um – mainly because we don’t have anything that can – besides treating depression or anxiety, and besides counseling with chaplaincy and social work and, you know, the physician themselves. Like, sometimes, if there’s still distress on top of all that, there’s nothing else – its hard – I don’t know if there’s any, I can’t think of anything else off the top of my head that we can truly offer somebody.”* *(S12, physician)*
	*“There are refractory cases where the distress is really significant and… doesn’t respond to these things like medications and talk therapy and a space to discuss it. I think there are certain cases where something else needs to be, you know, something else needs to be used… there are a number of cases where things are pretty refractory and not getting better with the standard treatment.”* *(S03, physician)*
**Theme 2: Palliative care providers ultimately see existential distress as a psychosocial-spiritual problem that evades medicalized approaches**	
Treatment of existential distress is de-emphasized within medical training and at times conflicts with standard medical culture	*“As a resident, oncology fellow, no. I don’t think I really learned a whole lot about existential distress, moral distress, spiritual distress until I was a palliative fellow.”* *(S01, physician)*
	*“That’s another barrier for you: they don’t feel comfortable when… they’re worried about uncovering the stress because they don’t know really what to do with it other than to refer to a social worker or someone else.”* *(S03, physician)*
	*“I think existential distress was what I felt most ill-equipped to handle. You know, that’s what we talk about least. And, you know, we have a solution for everything else: you have a medication for this and a medication for that.”* *(S11, nurse practitioner)*
	*“I think the lesson that I’ve learned about existential distress is that I always feel a little helpless in the face of it, because I don’t quite know how to fix it, or, you know I’m, even as a palliative care doc I’m like “ooh, there’s pain, I know what to do, I know what to do that will make that better!” And existential distress is much more murky than that.”* *(S05, physician)*
Primary treatment of existential distress is through meaning-focused approaches and interpersonal connection	*“The first thing that I’m going to recommend is actually a way to, um, for the patient to be able to express themselves and talk… Some of that really is us allowing the patient to kind of have a counseling and talking through those emotions, um, and I think that, honestly, being able to process is the best way to really deal with your existential stress because there’s no right – there’s no pill that I can prescribe that’s going to change what you’re going through. We have to see what your concerns are and then try to help reframe, if that makes sense.”* *(S09, physician)*
	*“But I think just like identifying it and naming it, being an empathic presence is helpful, and then kind of seeing what the patient feels that they need or what they may want. Because sometimes it’s just being heard, sometimes it’s like, “I want to, like, reconcile with my son now!” You know what I mean? Sometimes it’s like “I just want to talk about it, it’s been really hard.”* *(S01, physician)*
	*“My role. Yeah. So, I think it’s, you know just helping people just talk it through and really being fully present to listen to them. Um, and listen to the needs that are being expressed underneath and helping them sort of unpack it. And then helping them – and any chaplain will say this – having them come up with their own understanding and meaning around it.”* *(S17, chaplain)*
**Theme 3: Palliative care providers believe PAT hold promise for treating existential distress but that a stronger evidence base is needed**	
PAT is a new possibility for healing	*“I would say that if we are encountering a patient that is having, um, a lot of existential distress that seems to be unresponsive to our mainstream palliative care skills, in terms of like when we’re initially working through goals of care and building a rapport and relationship with them, um you know, if we rule out, for example, also depression or anxiety, other formal psychiatric illnesses – you know, because I wouldn’t want another psychiatric illness to go untreated – and so if we think it’s truly existential distress that is being unresponsive to our mainstream work within palliative care, I would love to have, um, a psycho-oncologist or, or a psychiatrist that’s available that, or that would help provide psilocybin for patients that we thought might be candidates.”* *(S06, physician)*
	*“So, for example, in California we’ve got the end of life option act where a patient can actually – as long as there’s a prognosis of 6 months or less – a patient can actually ask, you know, for the medications to, to end their own lives... So I could see this fitting in as a sort of a parallel offering, that, you know, if somebody is having extreme distress, and they don’t want to end their own life but they want to end that distress, and nothing else is working, that they should be able to say ‘yes, that’s, that’s a tool in your toolbox that I would be interested in trying.’”* *(S15, social worker/psychologist)*
	*“Sometimes the tools we have work well and people get there, but for [the rest] … this gives people the opportunity to look through a different lens, and to resolve some fears, and to figure out what it is – you know, kind of slow things down to figure out what it is that gives them meaning.”* *(S11, nurse practitioner)*
Stigma of psychedelics is still evident	*“I think I would worry about like the stigma of – I would just need, I would need to develop a pattern of how to introduce it to people. That would be my own work, and how I’m going to describe this to people. Because I can imagine that it would take a lot of folks who haven’t maybe read about it and you know are not maybe very medical, may be very health illiterate, and I could imagine them being like ‘Wait, what do you want me to do?’ and like ‘No way, that’s crazy, you sound like a crazy person.’ So I’d need to figure out how to talk about it.”* *(S05, physician)*
	*“I know some providers would probably be reluctant, because we don’t want to be known as ‘the psychedelic doctor’ – you know what I mean? Even with medical marijuana I think people feel concerned that we’re going to be seen as, like, the marijuana clinic or something like that – at least, here, and in some institutions.”* *(S01, physician)*
	*“I think there’s some people that just are afraid. You know, they’re afraid of side effects, and they’re afraid of like going down that sort of dark alley and not being able to come back from it.”* *(S15, social worker/psychologist)*
	*“You know then there’s the question of substance use, too, I guess, right? How much of this would be a… a continuation or lead to an escalation of substance abuse issues for someone who had a history of very poorly controlled substance use?”* *(S07, social worker/psychologist)*
Caution and safety are paramount	*“And then the other thing is it really does need: the people who are in the studies, but also who’d be using this in the future, they need to have a holding environment afterward, and some really skilled guidance – not just a couple of office appointments – to integrate, you know, what they’re exposed to inside.”* *(S13, chaplain)*
	*“My patients are all stressed, depressed, anxious. Some percentage have trauma histories, drug and alcohol histories, social support issues, so they’re fragile; many of them are pretty fragile. So I guess that’s where some of my reservations come from. I would certainly not want to introduce something that could, you know, further undermine their fragile psychological wellbeing. It’s good to know that the subjects have been rigorously screened, or psychologically screened for these things – that would be really appropriate moving forward.”* *(S07, social worker/psychologist)*
	*“I think that we also need to think about the guardrails, um, to place around agents that have this kind of potency to ensure that it’s the – that it’s properly trained people that are providing it in a safe setting. And that it’s, and that the patient is receiving longitudinal care from people that are attentive to existential distress, so that the patient is embedded within a psychologically-informed group like a palliative care group, you know, receiving ongoing care through palliative care or ongoing care through psycho-oncology.”* *(S06, physician)*
Further evidence and information sharing are needed before palliative providers can endorse PAT	*“I guess I would like to – it would be really great to see more research, with the data, I guess, would really help people like me feel more comfortable in its use, broader use. I think there should be more research, I think that would – that’s a good thing.”* *(S07, social worker/psychologist)*
	*[I]t would be wonderful if we could be trained, um, maybe a little bit more specifically – and it’s not to say that every single provider, necessarily, has to be trained, but, you know, like having a couple of specialists, right, on the teams that can help – either help with some of the decision-making, um, or helping to gauge, you know, who, who would be, who would this best be appropriate for.* *(S16, nurse practitioner)*
	*“So I think I would tend to try, you know, the, the sort of main-line, mainstream therapies first, um, and only go to the psychedelic if nothing else was working.”* *(S15, social worker/psychologist)*
**Theme 4: Because PAT do not currently fit existing models of existential distress treatment, barriers remain**	
There is no clear narrative of when, where, and for whom PAT should be offered	*“I could see it for people who have a great deal of anxiety – and people who actually are bringing a lot of their own, um, psychiatric issues into play and into this: I think it could really help, help them through a lot of different – in a lot of different ways.”* *(S19, social worker/psychologist)*
	*“I’ve had some patients that have had such trauma in their lives and some when they were young people, and they’ve never been able to get past that. And then, you know, when you get into an end of life situation everything gets heightened and it pulls back all these old painful memories and they can blow up out of proportion. And I think it is, it needs extreme measures. And from what I’ve read the psychedelic medication is possibly the only thing that would really help.”* *(S15, social worker/psychologist)*
	*“[The ideal patient for PAT is] someone who is relatively high-functioning, intelligent, verbal, good support system – good history of psychological functioning – has maybe had some experience with psychedelics in the past, so they have a sense of what to expect – had a positive experience in the past…someone with those general characteristics.”* *(S07, social worker/psychologist)*
	*“I can think of at least one person who I wish I could [offer PAT], um, but again unfortunately that patient has some – has contraindications that I wouldn’t be able to do it with. I wish that I could do it for that patient. So this has been someone who I’ve been working really hard with, and, um yeah, a lot of existential distress…But, again, there’s a mental health history that I’m unable to do so.”* *(S09, physician)*
Appeal of and access to PAT are not universal and cultural barriers remain	*“[T]here’s some people, I know for sure, there’s some people whose faith would say, ‘You are opening the door to evil. Like anything could walk right in then when you allow your mind to go like that with a chemical. It could be good, it could be angelic, it could be God, it could be positive. It could also be evil. And you know, like, there’s no way to know.’ There’s definitely that strain of faith.”* *(S17, chaplain)*
	*“But it’s hard to imagine like an institution who would need to spend 6 h with one like one patient. That would be great, I’d love that, I just can’t envision it happening in the real world.”* *(S05, physician)*
	*“I’m not sure here in the Deep South if you would have the same receptivity as you might in other, um, regions.”* *(S18, nurse practitioner)*
	*“Well, keep in mind I’m in New York City, so I’m in a big urban center, and it really has to do with my demographics. My younger patients I’d say – like 45 and under – I’d say maybe, like, 40%, like they’re more interested and open. And maybe some of my, like some of my older patients – like 60s, 70s, or some of those that have had experiences, you know, when they were younger, with experimenting. But it depends on what environment I’m working in, and what community. And definitely the – different demographics are more interested: I’d say especially the younger patients.”* *(S11, nurse practitioner)*

#### Theme 1: existential distress is a common experience that is frequently insufficiently treated within the current treatment framework

All participants identified existential distress as an important and relevant concept for their clinical palliative care work.

Respondents described existential distress as commonly arising both in the anticipation of dying as well as in response to changes in physical and psychosocial capabilities earlier in the course of illness. Existential distress was described as a disruption of, or challenge to, pre-existing sources of meaning or valued identities. Respondents noted a relationship between existential distress and psychiatric illness, though made a clear distinction between the two.

The absence of relationships or adequate social support was linked to greater likelihood of a patient meeting difficulty in resolving existential distress. However, concern for unmet emotional, relational, or financial needs for family members was also seen as a source of distress, and LTI was noted to give rise to existential distress through the exacerbation of prior traumas and unresolved interpersonal conflicts.

Respondents also described care of patients’ families as a critical part of their clinical role, and noted challenges to identity and meaning among caregivers for those with LTI. Respondents identified their own existential distress as an occupational hazard of working with patients at the end of life.

Refractory distress was reported across all types of clinical settings. Factors associated with refractory cases included late referral to palliative care, pre-existing psychiatric illness or trauma history, and young age, especially when the patient was a young parent.

Respondents, especially those from smaller hospitals or outpatient groups, pointed to barriers including limited time for visits, absence of specialty interventions and insufficient staffing to meet patient needs. Patient resource limitations were cited as a barrier to effective treatment in all settings.

#### Theme 2: palliative care providers ultimately see existential distress as a psychosocial-spiritual problem that evades medicalized approaches

All respondents endorsed existential distress treatment as falling within their scope of practice, with specific roles and degree of involvement differing across professional disciplines.

Respondents described existential distress as often overlooked or treated with discomfort by medical practitioners, especially primary consulting teams and oncologists. Physician and advanced practice nursing respondents reported an absence or paucity of training on existential distress treatment prior to specialization in palliative care. Palliative care approaches to existential distress were described as contrasting with a general medical culture focused on biological models of diagnosis and treatment.

Participants endorsed therapeutic interpersonal techniques as the primary intervention for existential distress, including active empathic listening and nonjudgmental exploration of patients’ distress. More complicated cases were described as requiring specialist intervention with providers of spiritual care or psychotherapy, of which the most common types were meaning-centered, cognitive behavioral, and mindfulness-based psychotherapies. Helping patients repair or build new personal relationships was seen as integral to reducing existential distress.

#### Theme 3: palliative care providers believe PAT hold promise for treating existential distress but that a stronger evidence base is needed

Respondents expressed interest in expansion of research and clinical access to PAT. However, not all were enthusiastic proponents and reservations or skeptical attitudes were common.

PAT was identified as a potentially powerful addition to the palliative care “toolkit” and respondents described PAT as facilitating meaning-making by allowing patients new perspectives through which to reframe their existential struggle. Respondents endorsed the use of PAT through compassionate use provisions, describing PAT as providing hope for patients experiencing refractory existential distress. Respondents saw PAT as an alternative to controversial interventions for end-of-life distress, such as palliative sedation or physician aid-in-dying.

At the same time, participants identified the current evidence base as insufficient and cited a need for further PAT research and greater education on PAT within palliative care departments before they could feel confident in its use. Participants described PAT as late-line interventions to be used only after other therapies had failed.

Participants also expressed concerns about stigma for patients receiving PAT, as well as fears about stigmatization from other medical providers for providing PAT. Participants reported concerns that use of PAT would trigger relapse for patients with substance use disorders, and expressed worries about lasting psychological harm from dysphoric psychedelic experiences. Attitudes were more negative toward LSD and positive toward psilocybin and ketamine.

Participants expressed confidence in the safety of PAT in properly controlled settings. Respondents emphasized key safety considerations for PAT, including rigorous screening for cardiac disease or history of mania or psychosis, skilled supervision during medication-facilitated sessions, and longitudinal psychotherapy follow-up to integrate these experiences.

#### Theme 4. Because PAT do not currently fit existing models of existential distress treatment, barriers remain

Providers struggled to identify clearly how PAT could fit into the current treatment paradigm, with fundamental questions unanswered regarding for whom PAT might be an appropriate and accessible treatment.

There was no clear consensus around the relative benefits of delivering PAT to patients early in their course of LTI, later while admitted as inpatients, or even while on hospice. Respondents similarly failed to present clear agreement about which palliative care patients should be considered for PAT. Some described psychiatric and trauma histories as important indicators of patients who might benefit most from PAT, while others expressed concern regarding PAT for patients with any psychiatric comorbidity.

Providers described interest in PAT as concentrated within specific sociocultural groups, such as younger patients in urban settings. Respondents estimated greater interest in West Coast and Northeastern states than in the Southern US. Participants identified patient groups for whom PAT might not be appealing, such as particular religious groups, and described PAT as cost-prohibitive and likely to exclude poor and underserved communities.

## Discussion

The present study reports attitudes toward the treatment of existential distress using PAT within a representative sample of palliative care professionals. PAT were seen as holding promise to improve the treatment of existential distress within palliative care settings, especially in refractory cases. Overall, respondents identified further research and outreach as necessary before PAT can be expanded in palliative care settings, and identified several unresolved barriers to implementation of PAT in palliative care settings.

### Broadening the team

One of the most striking features of these interviews was the emphasis on interdisciplinary collaboration within palliative care, a central cultural tenet of the field. Notably, both subthemes that failed validation touched on questions of role boundaries, and likely were rejected due to perceived violations of this core value.

“*Physicians and advanced practice nurses have limited roles in the treatment of existential distress as compared to chaplains and social workers” (Theme 2)* was drawn from descriptions of medical providers serving primary screening roles and triaging cases of existential distress to spiritual care or mental health care providers. However, the language of this subtheme was poorly considered, and respondents in the validation sample emphasized a team-based approach to the treatment of existential distress in which distinct roles are equally valuable.

Similarly, the second unvalidated subtheme dealt with the possibility of PAT marking a greater involvement of psychiatrists and other mental health practitioners in existential distress care: *“PAT would mark a significant change in how and by whom existential distress is treated” (Theme 4).* Feedback on this subtheme suggests that while respondents agree PAT would require greater involvement of psychiatrists and other mental health providers, palliative care providers would prefer to expand the umbrella of palliative care rather than referring cases to outside collaborators. This reflects the growing field of psycho-oncology and the greater incorporation of psychiatric and mental health providers into the multidisciplinary palliative care team. Furthermore, respondents reported strong interest in obtaining training in PAT within palliative care departments, despite no explicit question directed at this topic.

This study, by exploring current standards of care and attitudes toward PAT in a sample reflective of the interdisciplinary culture of palliative care, improves upon and further contextualizes recent investigations of attitudes toward PAT among palliative care providers. Those studies, which addressed perspectives of a diverse group of experts in palliative care, oncology and PAT [28] and a small sample of palliative care providers from a single department [29], described attitudes that are echoed in our broad sample of palliative care workers. Concerns regarding negative psychological experiences, hopes for a transformative new treatment, and an emphasis on larger trials to better understand risks and contraindications of PAT appear across all groups. The present study grounds discussions further in fundamental questions of how existential distress is currently treated, offering a perspective on PAT that is informed by contemporary practice and described gaps in care.

### A narrow gap

Expansion of research into PAT in palliative care settings will be facilitated by the clear identification of a target population. While some participants reported an ideal of offering access to PAT for all patients facing life-threatening illness, PAT were commonly seen as intensive treatments indicated only after conventional methods have failed. Perceptions of PAT as late-line therapies may be reflective of stigma around psychedelics and thus subject to change with greater education about PAT. Still, many participants saw conventional treatments of existential distress as largely adequate, which invites us to consider the exact treatment gap PAT might fill.

The greatest need for PAT may be within populations especially likely to suffer from refractory existential distress, such as younger adults or patients with significant trauma histories. Further research would be best directed at identifying risk factors for poor response to conventional psychotherapy and spiritual counseling. Alternatively, referral to PAT could be triggered by specific shifts in the course of treatment associated with significant unmet existential needs, such as cancer recurrence, hospice enrollment, or transition from standard cancer therapies to phase I clinical trials [35].

Notably, the characteristics of patients with refractory existential distress overlapped with those of patients deemed too ill or unstable to be safely considered for PAT. Further studies are needed to clarify which psychiatric comorbidities are contraindications for PAT. Without this, the great challenge is that the window of opportunity – patients sick enough for PAT but not sick enough to be at risk of destabilization – may be narrow.

Similarly, if treatment for existential distress is more limited in less-resourced settings, PAT is not well-situated to amend this treatment gap. Even if PAT are covered by insurance, the significant time demand of treatments may perpetuate problems of power and access that continue to plague American medicine, with marginalized groups unable to benefit equitably from these therapies. Research efforts to date have failed to adequately include diverse samples, with patients of color underrepresented in PAT studies [36]. Further research is needed to determine the causes of and solutions for these disparities.

### Pathways to integration

Respondents in our sample described meaning-enhancing interpersonal interventions, including both spiritual care and psychotherapeutic frameworks, as the core of conventional existential distress treatment. That meaning-making specifically mediates benefits of some conventional treatments [37] underscores respondents’ perceptions of PAT as an intervention with unique capacities to facilitate meaning-making. The capacity of psychedelic interventions to enhance personal meaning and spiritual significance is well established [38, 39]. In clinical studies, the majority of participants rate their sessions as among the top five, or even the single most, meaningful or spiritually significant experience of their lives [38] — a finding demonstrated to persist among survivors in a small, 4.5-year follow-up of PAT for cancer-related distress [40].

Our findings suggest that PAT might be most easily integrated into palliative care practice if delivered in a manner consistent with current first-line meaning-enhancing approaches of psychotherapy and spiritual counseling. As researchers explore synergies between PAT and specific therapeutic modalities in other clinical contexts [41, 42], efforts to more explicitly integrate existentially-oriented therapies such as Meaning-Centered Psychotherapy [10], Dignity Therapy [11], or Managing Cancer and Living Meaningfully (CALM) [12] into preparatory and integration sessions might strengthen its efficacy as a treatment for existential distress. In order to integrate PAT into current approaches to existential distress, support of spiritual care providers in addition to secular mental health providers is paramount, and PAT researchers should include spiritual care providers within treatment paradigms when possible.

Broader collaboration between PAT and faith traditions will not be without challenges. While psychedelic agents are important sacraments for some spiritual communities [43, 44], respondents suggested the possibility that other religious and cultural groups may reject PAT due to concerns of incompatibility with established doctrine. However, participants stressed the need for individualized, patient-centered approaches to selecting therapies for existential distress, and we echo their proscriptions against ruling out inclusion by any particular group.

These findings also suggest the importance of maintaining emphasis on PAT’s meaning-enhancing effects. Psilocybin’s FDA designation as a breakthrough therapy for major depressive disorder [45, 46], while promising for the general psychiatric population, is not without pitfalls. Viewing psilocybin as a purely biological intervention that functions to boost subjective mood, as with SSRIs and ketamine, risks eliding the profound spiritual and personal meaning that patients attribute to medication-facilitated sessions and that may mediate benefits [38]. Equating PAT’s clinical benefit with the reduction of mood symptoms risks solidifying an understanding of PAT that ignores its immense potential to catalyze meaning-making for patients with LTI.

### Stigma and barriers

Positive and stigmatizing views toward PAT were both common, often co-occurring within the same interview. This demonstrates the complicated way in which providers integrate new information about the therapeutic potentials of PAT with decades-old understandings of psychedelics as dangerous and illegal agents. While concerns about potential risks of PAT for patients with cardiac comorbidities echoe the exclusion criteria of recent studies, respondents also voiced concerns that do not track with recent evidence. Fears that PAT might trigger relapses among patients with substance use disorders contrasts with observations that psychedelics decrease patterns of problematic substance use in naturalistic settings [47–49]; PAT are being trialed as treatments for addiction [50, 51]. Similarly, concerns about persistent negative psychological effects are unsupported by the evidence. While acute anxiety during PAT is not uncommon [52, 53], there is no record of serious adverse events during modern, setting-controlled studies [54], and even those who experience transient psychedelic-related dysphoria or anxiety often go on to report these experiences as ultimately beneficial [53–55].

These examples highlight the importance of education to dispel longstanding misconceptions regarding carefully monitored psychedelic use and promote data-driven understandings of the risks associated with PAT. While the present study was underpowered to assess variations in knowledge and attitudes toward PAT across professional classes, further survey-based research should seek to better classify these patterns, as has been demonstrated in larger samples of psychiatrists [56] and psychologists [57].

### Limitations and strengths

Convenience and snowball recruiting methods may have resulted in bias toward the cultures and institutional orthodoxies of included sites. Attitudes may be culturally specific and not necessarily generalizable outside the US. The sample of respondents was skewed toward younger and less experienced clinicians, who may be more likely to have positive views toward psychedelic therapies [56]. While the sample demonstrated minimal racial diversity, with all but one participant identifying as White/Caucasian, this may reflect broader trends in the palliative and hospice care workforce. The major strengths of this study are its broad inclusion of palliative care professional disciplines and emphasis on existing treatments for existential distress. Grounding discussions of PAT in the current standard of care offers a practically-informed model for integration into palliative care.

## Conclusions

Palliative care providers describe existential distress as a common source of suffering for patients with LTI. Current treatments emphasize enhancement of sources of meaning and rely on interdisciplinary coordination. Clinicians view PAT as promising treatments for refractory existential distress, though concerns regarding access and exclusionary criteria currently limit their potential scope. Further research and education regarding psychedelic interventions are needed before PAT can be more widely adopted in palliative care settings, especially to address safety concerns and clarify a target population. Close collaboration with spiritual care and mental health providers and adaptations of PAT to existing meaning-focused approaches will facilitate integration into current practice. Educational outreach should address misconceptions regarding risks of substance use and psychological harm. Broader access to PAT research and greater diversity of study samples will improve generalizability and promote equitable treatment outcomes.

## Supplementary Information

**Additional file 1.** Semi-structured interview protocol.

## Data Availability

The data generated by this project are not publicly available due to the sensitive nature of responses and possibility of participant identification. De-identified data are available from the study authors on reasonable request.

## Abbreviations

LTI: Life-threatening illness
MDMA: 3,4-Methylenedioxymethamphetamine
PAT: Psychedelic-assisted therapies
