# Supplementary material for: Palliative care provider attitudes toward existential distress and treatment with psychedelic-assisted therapies
Source: BMC Palliat Care. 2021 Dec 20;20:191. doi: 10.1186/s12904-021-00889-x (PMC8690623; doi:10.1186/s12904-021-00889-x)
Supplement: Supplementary file 1 — Additional file 1. Semi-structured interview protocol. [file 12904_2021_889_MOESM1_ESM.docx]

**Semi-structured interview protocol**

1. Do you treat patients facing life-threatening illness? How many in the past year?
2. How do you see your role with regards to the existential distress of patients facing life-threatening or life-limiting illness?

Have you received any specific training for treating these symptoms?

What resources, treatments, or medications do you have at your disposal?

1. How well do you think current practices treat the existential distress of patients facing the end of life or the potential end of life?

Is there anything that you think is lacking or that needs to change?

1. What have you heard regarding the use of psychedelic-assisted therapy to treat existential distress in the context of life-threatening or life-limiting illness?

Do you think psychedelics could be used safely in a medical context?

1. Were such treatment legally available, would you prescribe or recommend it for your patients?

If so, in what circumstances? What concerns would you have? Would there be patients

or situations where you would be more or less likely to advise treatment with

psychedelic-assisted therapy?

If not, why not? What might make you more likely to consider its use?

1. In thinking about the future of palliative care, what place, if any, do you think psychedelic-assisted therapies should have in the treatment of patients facing existential distress in the setting of life-threatening or life-limiting illness?

What developments in research, clinical, or societal factors might change your mind, in

either direction?
